# Carotenoids Do Not Protect Bacteriochlorophylls in Isolated Light-Harvesting LH2 Complexes of Photosynthetic Bacteria from Destructive Interactions with Singlet Oxygen

**DOI:** 10.3390/molecules26175120

**Published:** 2021-08-24

**Authors:** Zoya K. Makhneva, Maksim A. Bolshakov, Andrey A. Moskalenko

**Affiliations:** Institute of Basic Biological Problems RAS, 142290 Pushchino, Russia; zmakhneva45@rambler.ru (Z.K.M.); lfbv22@jmail.com (M.A.B.)

**Keywords:** purple photosynthetic bacteria, light-harvesting complex LH2, bacteriochlorophyll, carotenoid, oxidation, singlet oxygen, generation of singlet oxygen by carotenoids

## Abstract

The effect of singlet oxygen on light-harvesting (LH) complexes has been studied for a number of sulfur (S^+^) and nonsulfur (S^−^) photosynthetic bacteria. The visible/near-IR absorption spectra of the standard LH2 complexes (B800-850) of *Allochromatium* (*Alc*.) *vinosum* (S^+^), *Rhodobacter* (*Rba*.) *sphaeroides* (S^−^), *Rhodoblastus* (*Rbl*.) *acidophilus* (S^−^), and *Rhodopseudomonas* (*Rps*.) *palustris* (S^−^), two types LH2/LH3 (B800-850 and B800-830) of *Thiorhodospira* (*T*.) *sibirica* (S^+^), and an unusual LH2 complex (B800-827) of *Marichromatium* (*Mch*.) *purpuratum* (S^+^) or the LH1 complex from *Rhodospirillum* (*Rsp*.) *rubrum* (S^−^) were measured in aqueous buffer suspensions in the presence of singlet oxygen generated by the illumination of the dye Rose Bengal (RB). The content of carotenoids in the samples was determined using HPLC analysis. The LH2 complex of Alc. vinosum and *T*. *sibirica* with a reduced content of carotenoids was obtained from cells grown in the presence of diphenylamine (DPA), and LH complexes were obtained from the carotenoidless mutant of *Rba*. *sphaeroides* R26.1 and *Rps*. *rubrum* G9. We found that LH2 complexes containing a complete set of carotenoids were quite resistant to the destructive action of singlet oxygen in the case of Rba. sphaeroides and *Mch*. *purpuratum.* Complexes of other bacteria were much less stable, which can be judged by a strong irreversible decrease in the bacteriochlorophyll (BChl) absorption bands (at 850 or 830 nm, respectively) for sulfur bacteria and absorption bands (at 850 and 800 nm) for nonsulfur bacteria. Simultaneously, we observe the appearance of the oxidized product 3-acetyl-chlorophyll (AcChl) absorbing near 700 nm. Moreover, a decrease in the amount of carotenoids enhanced the spectral stability to the action of singlet oxygen of the LH2 and LH3 complexes from sulfur bacteria and kept it at the same level as in the control samples for carotenoidless mutants of nonsulfur bacteria. These results are discussed in terms of the current hypothesis on the protective functions of carotenoids in bacterial photosynthesis. We suggest that the ability of carotenoids to quench singlet oxygen (well-established in vitro) is not well realized in photosynthetic bacteria. We compared the oxidation of BChl850 in LH2 complexes of sulfur bacteria under the action of singlet oxygen (in the presence of 50 μM RB) or blue light absorbed by carotenoids. These processes are very similar: {[BChl + (RB or carotenoid) + light] + O_2_} → AcChl. We speculate that carotenoids are capable of generating singlet oxygen when illuminated. The mechanism of this process is not yet clear.

## 1. Introduction

Singlet oxygen is capable of oxidizing many targets in the cell including proteins, pigments, lipids, etc. Singlet oxygen is formed when an oxygen molecule receives energy from an excited photosensitizer molecule. In the case of purple bacteria, one such photosynthesizer is bacteriochlorophyll (BChl), which, in some cases, can pass from a singlet state to a long-lived triplet state and then interact with oxygen to form singlet oxygen (^1^O_2_*):

BChl + hν → ^1^BChl*→ ^3^BChl* + O_2_ → BChl + ^1^O_2_* (1)

Cells are faced with the task of preventing the formation of singlet oxygen, which can be solved in two ways: 1—the quenching of BChl triplets and 2—the quenching of singlet oxygen. In both cases, the main role is played by carotenoids (Schemes (2) and (3)).

BChl + hν → ^1^BChl*→ ^3^BChl*→ BChl + ^3^Car* → Car + heat (2)

^3^BChl* + O_2_→ BChl + ^1^O_2_* → ^1^O_2_* + Car → O_2_ + ^3^Car* → Car + heat (3)

A hypothesis was first proposed for the protective function of carotenoids in vivo [1] based on the results of growing control cells of *Rhodobacter* (*Rba*.) *sphaeroides* and cells of a blue-green carotenoidless mutant in light under anaerobic or aerobic conditions. In an accompanying note, this hypothesis was supported [2]. At present, such a protective function is generally accepted. In the overwhelming majority of subsequent works, the interaction of BChl and carotenoids was studied in detail (see: [3,4,5,6,7]).

The processes described above take place in specialized bacterial nanostructures—pigment–protein complexes in which BChl and carotenoids are localized. Three types of complexes are usually distinguished: the reaction center (RC), light-harvesting LH1 complex, and the peripheral light-harvesting LH2 complex. The LH2 complex consists of 8 (*Rhodospirillum* (*Rsp*.) *molischianum*, *Ectothiorhodospira* (*Ect*.) *haloalkaliphila*), 9 (*Rba*. *sphaeroides*), or 12 (*Allochromatium* (*Alc*.) *vinosum*) pairs of low-molecular-weight polypeptides (α and β). BChl molecules in the LH2 complex form symmetric ring aggregates absorbing near ~800 and ~850 nm, respectively. BChl850 molecules are localized between the α- and β-polypeptide rings, and the BChl800 (“monomer”) is on the outer side of the LH2 complex. Carotenoids are located between the α- and β-polypeptide rings in the so-called “carotenoid pockets”, so that their polyene chain passes perpendicular to the membrane and interacts with BChl molecules in the corresponding clusters (BChl800, BChl850) and amino acid residues of both polypeptides [8,9,10,11,12,13]. They are C-40 molecules and their synthesis begins with the precursor phytoene, from which neurosporene (the spheroidal pathway) or lycopene (the spirilloxanthin pathway) are formed through several desaturation steps, respectively. This is followed by a series of stages of desaturation, saturation, etc., as well as the introduction of various groups (hydroxyl, methyl, etc.) and, as a result, a system of conjugated double bonds is formed in the carotenoid molecule, which is capable of dissipating the energy received from BChl in the form of heat [14].

While the interaction of BChl with carotenoids has been studied quite well, in the case of singlet oxygen (the BChl–carotenoids–singlet oxygen system), there are some “white spots”. It is not entirely clear where singlet oxygen can form. Its release with a quantum yield of 0.03 ± 0.005 has been shown only for isolated bacterial RC [15,16], and for light-harvesting complexes, there are still no such measurements. Perhaps these complexes can take part in this process, since wild *Rba*. *sphaeroides* 2.4.1 generated a small amount of singlet oxygen under high-light conditions, and it was considered a signaling molecule for the expression of a number of genes in the cells of these bacteria [17]. It is still not clear how efficiently carotenoids are able to quench singlet oxygen, whether BChl in vivo needs protection from singlet oxygen, or what types of molecules (BChl or carotenoids) are the source of singlet oxygen generation in bacterial cells. To answer some of these questions, it is necessary to compare the effect of singlet oxygen on control samples and samples with a reduced carotenoid content or without carotenoids. With the complete inhibition of the biosynthesis of carotenoids (mutation in the CrtB and CrtI genes), the assembly of the LH2 complex is interrupted, and the membranes contain only the LH1 complex type. For example, the R26 mutant of *Rba*. *sphaeroides* contains the LH1 complex and R26.1 mutant—the pseudo LH1 complex (modified LH2 complex without BChl800) [1,3,4,18]. It is currently not possible to obtain mutants with complexes that would contain 10% or 50% of the control content of carotenoids using genetic methods. Our approach using an inhibitor allows us to solve this problem and obtain complexes with different carotenoid content (in the range of 1.5 ÷ 100%) [19,20,21,22]. In this work, we studied the effect of singlet oxygen on BChl oxidation in light-harvesting LH2 complexes having different compositions and carotenoid contents from sulfur and nonsulfur bacteria, and we also investigated the effect of light absorbed by the carotenoids on BChl photooxidation in these complexes. These complexes represent a convenient model system for studying the processes of interaction of singlet oxygen with pigments. It is clear that they are less important for sulfur bacteria, which are anaerobes or strict anaerobes, but significant for nonsulfur bacteria, which can grow at different oxygen concentrations in the growing medium. A wide range of samples from different bacteria was specially taken to show that the effects we observed are of general biological significance.

## 2. Results and Discussion

In this work, we simulated the conditions in which the system is at a high concentration of singlet oxygen using the Rose Bengal (RB) dye, which efficiently generates singlet oxygen under illumination. In fact, we simulated the left side of Equation (3)—“^3^BChl* + O_2_ → BChl + ^1^O_2_*”, in which BChl triplets interact with oxygen to form singlet oxygen. This system allowed us to check two aspects of the problem: (1)—Can BChl be oxidized by ^1^O_2_* in the presence of carotenoids? (2)—How effectively do carotenoids quench ^1^O_2_*, preventing BChl oxidation? The RB concentration (50 μM) was selected so that the effect of ^1^O_2_* for 30 min was similar in the effect to BChl bleaching in blue-green light.

### 2.1. Interaction of Singlet Oxygen with BChl in LH2 Complexes with a Full Set of Carotenoids

**Figure 1:**
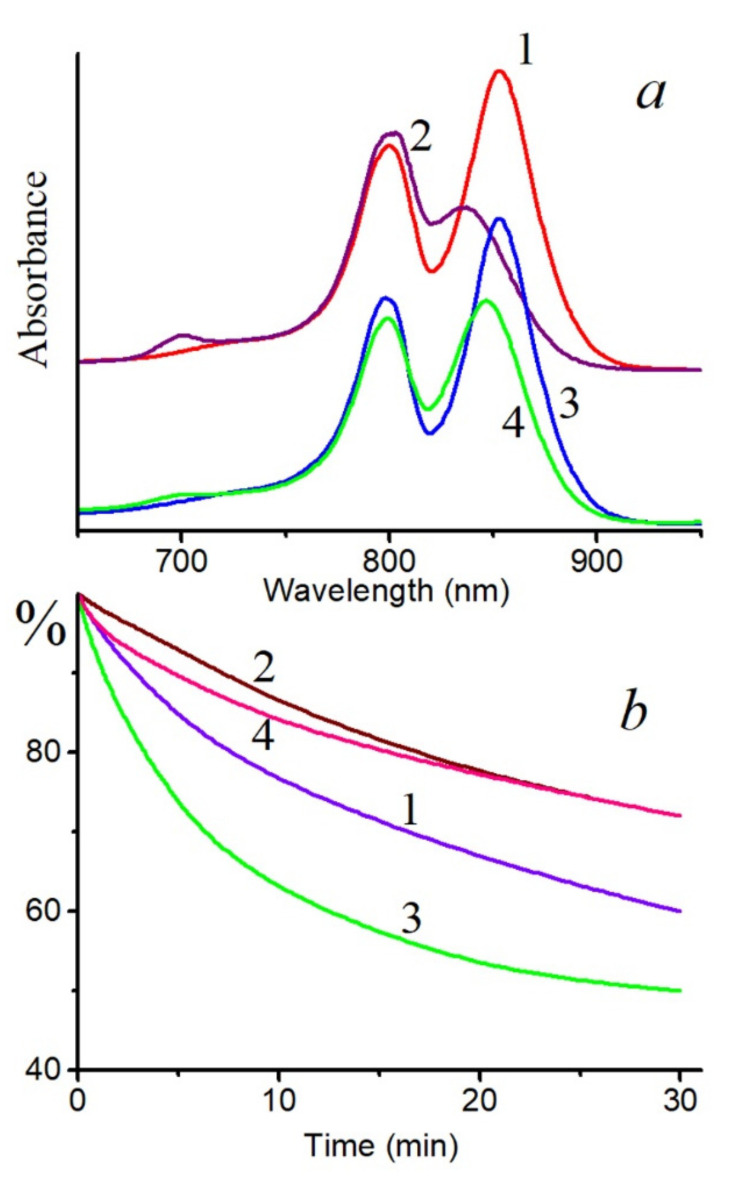
(**a**), Absorption spectra of control (1, 2) and diphenylamine (DPA) (3, 4) LH2 complexes of *Alc*. *vinosum* before (1, 3) and after illumination for 30 min in the presence of 50 μM RB (2, 4); (**b**), bleaching curves of BChl850 in the control (1, 3) and DPA (2, 4) complex LH2 from *Alc. vinosum* in the presence of 50 μM RB (1, 3) and under illumination with blue light (2, 4).

**Figure 2:**
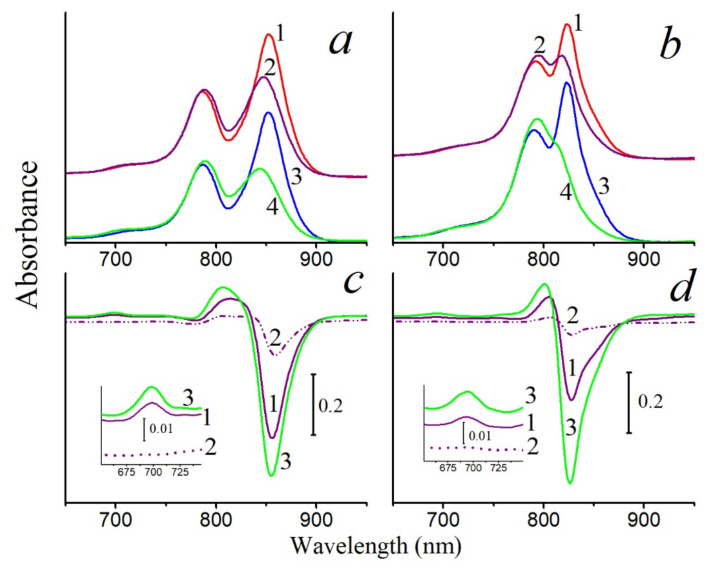
(**a**), Absorption spectra of the control LH2 complex of *T*. *sibirica* before (1, 3) and after illumination for 30 min in the presence of 50 μM RB (2) and blue light (4); (**b**), absorption spectra of the control LH3 complex of *T*. *sibirica* before (1, 3) and after illumination for 30 min in the presence of 50 μM RB (2) and blue light (4); (**c**)—differential absorption spectra of the LH2 complex of *T*. *sibirica* under illumination: 1—“30 min in the presence of 50 μM RB minus 0 min”; 2—“30 min in the absence of 50 μM RB minus 0 min”; 3—“30 min illumination with blue light minus 0 min”; Inset: Differential absorption spectra in the region of 3-acetyl-chlorophyll (designations are the same as in Figure 3c); (**d**)—Differential absorption spectra of the LH3 complex of *T*. *sibirica* after illumination: 1—“30 min in the presence of 50 μM RB minus 0 min”; 2—“30 min in the absence of 50 μM RB minus 0 min”; 3—“30 min with blue light minus 0 min”; Inset: differential absorption spectra in the region of 3-acetyl-chlorophyll (designations are the same as in Figure 3d).

A decrease in the BChl850 band of the LH2 complex from *Alc*. *vinosum* (full spectrum without RB: Figure S1) under the action of singlet oxygen was noted, while the BChl800 band slightly increased due to a shift of the first band to shorter wavelengths. At the same time, a band of the BChl oxidation product 3-acetyl-chlorophyll (AcChl) appeared near 700 nm (Figure 1a). Similar changes in the absorption spectrum of the LH2 complex from *Alc*. *vinosum* were seen during chemical oxidation of BChl [23]. The long-wavelength absorption band also decreased in the LH2 and LH3 complexes from *Thiorhodospira* (*T*.) *sibirica* under the action of singlet oxygen, and the formation of the BChl oxidation product was less pronounced and was revealed only in the difference spectrum (Figure 2). It should be emphasized that, if RB is not included in the system, then formation of the BChl oxidation product does not occur under the action of the corresponding light, and the long-wavelength absorption band of BChl decreases by 26 and 15% in the LH2 and LH3 complexes, respectively. In the LH2 complex of *Alc*. *vinosum*, which is most sensitive to the action of singlet oxygen, this parameter does not exceed 10%.

**Figure 3:**
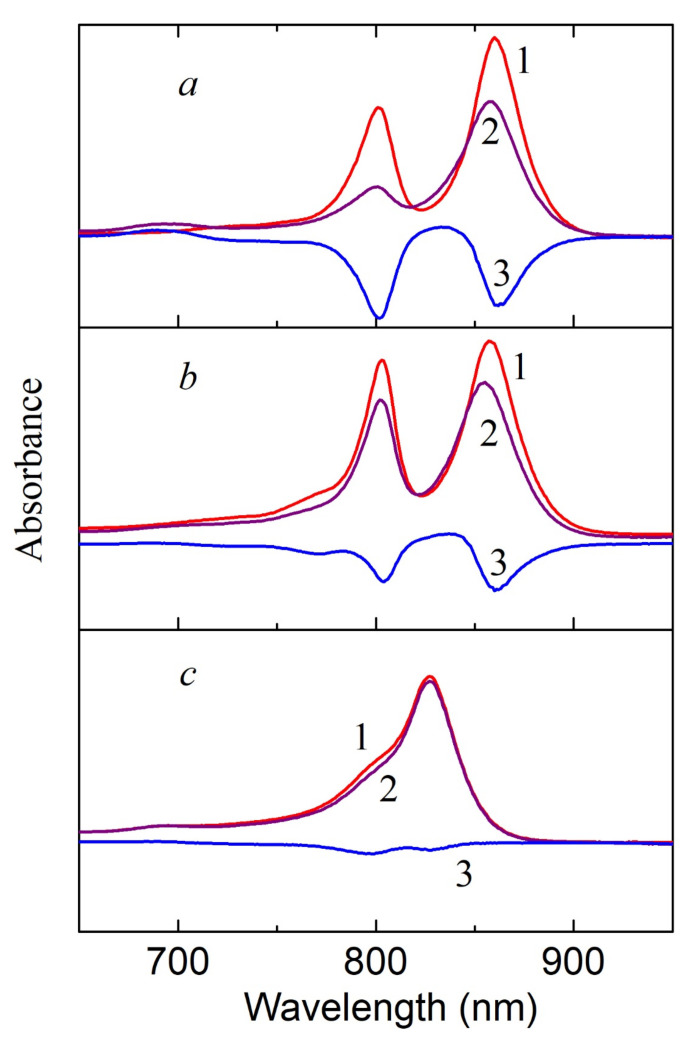
Absorption spectra of LH2 complexes from *Rbl*. *acidophilus* (**a**), *Rps*. *palustris* (**b**), and *Mch. purpuratum* (**c**) before (1) and after (2) 30 min illumination in the presence of 50 μM RB. Differential absorption spectrum “30 min illumination in the presence of 50 μM RB minus 0 min” (3).

**Figure 4:**
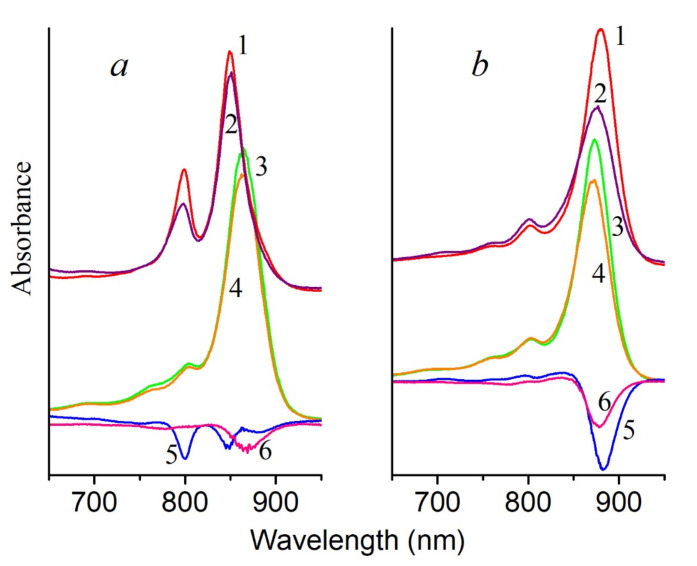
Absorption spectra of LH2 complexes from *Rba*. *sphaeroides* (**a**) and LH1 complexes from *Rps*. *rubrum* (**b**) 1, 2, 5—with carotenoids (wild strain); 3, 4, 6—without carotenoids (carotenoidless mutant R-26 or G9 strain, respectively): before (1, 3) and after (2, 4) 30 min illumination in the presence of 50 μM RB. Differential absorption spectrum “30 min illumination in the presence of RB minus 0 min” (5, 6).

BChl molecules from both clusters (BChl800 and BChl850) are oxidized in LH2 complexes from nonsulfur bacteria under the action of singlet oxygen (Figure 3 and Figure 4a). In this case, the formation of AcChl was seen only for *Rhodoblastus* (*Rbl*.) *acidophilus*, and its maximum was located at 693 nm. This product was absent from the absorption spectra of *Rhodopseudomonas* (*Rps*.) *palustris*, *Marichromatium* (*Mch*.) *purpuratum*, and *Rba*. *sphaeroides* (Figure 3b,c and Figure 4a), which is possibly associated with the absence of BChl oxidation (Figure 3c) or its deeper oxidation (Figure 3b and Figure 4a) with the formation of colorless products, products with an open ring, etc. It was shown that, during BChl photooxidation under illumination in a solvent, up to 12 different substances are formed, including bacteriochlorins, chlorins, products with an open ring, and colorless products [24]. BChl from *Mch*. *purpuratum* and *Rba*. *sphaeroides* are the most resistant to this oxidant. The structure of the LH2 complex from *Mch*. *purpuratum* was recently reported with 2.4-Å resolution [25]. It was shown that the LH2 complex consists of seven pairs of α/β heterodimers and that there are two carotenoids, including okenone, with a χ-ring at one end for each heterodimer. The second carotenoid is localized on the outer side of the complex and is involved both in the additional pathway of energy transfer to BChl (BChl800) and in the formation of heterostructures from the LH2 complexes in the membranes. Note that this complex has an unusual absorption spectrum, and its resistance to the action of singlet oxygen requires additional study. Of the standard LH2 complexes of the B800-850 type studied in this work, the LH2 complex from *Rba*. *sphaeroides* was the most resistant to the action of singlet oxygen. This bacterium was studied in classical works [1,26].

Based on this stage of the work, it became clear that singlet oxygen, which is released when RB is illuminated with the appropriate light, is capable of penetrating in an aqueous medium to BChl molecules in LH2 complexes and oxidizing them. The LH2 complexes from different bacteria (sulfur and nonsulfur) with a full set of carotenoids differed from each other in the degree of the resistance of BChl to the action of singlet oxygen. All of these bacteria (with the exception of *Mch.* *purpuratum*) contain similar carotenoids, which differ in the number of double bonds and side groups. Many of them quench singlet oxygen in model systems with high efficiency [27,28,29]. Unfortunately, there is currently no assessment of how efficiently this function is performed in situ (in pigment–protein complexes). In this work, only a decrease (oxidation) of the absorption bands of BChl in the LH2 complexes was recorded. However, it is not possible to evaluate the efficiency of singlet oxygen quenching by carotenoids in the complexes.

The efficiency of singlet oxygen quenching by carotenoids in LH2 complexes can be estimated by comparing BChl oxidation: (a)—in LH2 complexes with a full set of carotenoids; (b)—in LH2 complexes with reduced carotenoid content; (c)—in carotenoidless complexes (mutants).

### 2.2. Interaction of Singlet Oxygen with BChl in LH2 Complexes with Low Content or without Carotenoids

Figure 4 shows the results of the effect of singlet oxygen on control and carotenoidless LH2 complexes from *Rba*. *sphaeroides* and LH1 complexes from *Rps*. *rubrum*. In fact, this experiment created the conditions that should exist in a carotenoidless mutant under strong light in the presence of oxygen: the formation of BChl triplets, their interaction with oxygen, and the generation of singlet oxygen. According to the general opinion, this process should lead to the oxidation of BChl by singlet oxygen. However, in the given case, the last stage is absent in the carotenoidless LH complexes: they are also stable in the presence of singlet oxygen as the control complexes. These data are in good agreement with our earlier results, which showed that BChl in the membranes of the carotenoid-free mutant *Rba*. *sphaeroides* R26.1 is more resistant to the action of singlet oxygen than BChl in control membranes with carotenoids [30]. Thus, based on these results, we can make the rather reasonable speculation that carotenoids in this case are not required to protect BChl from oxidation by singlet oxygen in LH complexes.

The second stage of this experiment, namely, determining the effect of the amount of carotenoids on the stability of BChl in LH2 complexes under the action of singlet oxygen, was carried out using LH2 complexes from sulfur bacteria. This was done for two reasons: first, the biosynthesis of carotenoids can be almost completely inhibited in these sulfur bacteria (this cannot be done in nonsulfur bacteria), and LH2 complexes with different carotenoid content were isolated by varying the concentration of the inhibitor (spectra and carotenoid content: Figures S1 and S2; Table S1); second, BChl in these complexes is easily oxidized by singlet oxygen even in the presence of carotenoids (Figure 1 and Figure 2).

In LH2 complexes from *Alc*. *vinosum* with little carotenoids (DPA samples), a slower oxidation of BChl850 was noted at the same BChl concentration as in the control (Figure 1a). The content of carotenoids in the DPA LH2 complex did not exceed 8–10%. A simple calculation shows that, if the control LH2 complex contains 12 carotenoids, then in LH2 complex from DPA-grow cells, their number does not exceed 1 pigment per complex. This follows from the observation that a decrease in the number of carotenoid molecules in the LH2 complex (from 12 to ≈1) slightly increased its resistance to the action of singlet oxygen. Similar results were obtained for LH2 and LH3 complexes from *T*. *sibirica* (Figure 4 and Figures S3 and S4): a decrease in the amount of carotenoids in one complex (according to our calculations, when it was decreased from 8 (control) to 7, 4-3, and 1 carotenoid per complex, respectively) the stability of BChl850/830 was increased in relation to the action of singlet oxygen by 18–27% for the LH2 complex and by 9–18% for the LH3 complex. Note that, according to the generally accepted opinion about the protective function of carotenoids, BChl in LH2 complexes with a reduced content of carotenoids or their complete absence (carotenoidless mutants) should be easily oxidized by singlet oxygen. Our data show that a decrease in the amount of carotenoids in the complexes increases their resistance to the action of singlet oxygen.

We used the simple native system (bacterial complex LH2/3), including two types of pigments (BChl and carotenoids) and polypeptides, which are able to absorb the energy of a quanta of sunlight, convert it into excitation energy, and then dissipate the latter in the form of radiant energy or heat. The addition of RB as a source of singlet oxygen to this system makes it possible to study the effect of the latter on the spectral characteristics of complexes, as well as to study other related processes. Earlier, we showed that singlet oxygen can diffuse into LH complexes in the membranes of purple bacteria, where it interacts with BChl and oxidizes the latter [30]. This scheme also works in the case of the LH2 complexes with carotenoids, in which BChl, depending on the type of bacteria, is oxidized with different efficiencies by singlet oxygen. The lifetime of the singlet oxygen, in this system, is probably sufficient for this process to occur. BChl850 in the LH2/3 complexes from the sulfur bacteria *Alc*. *vinosum* and *T*. *sibirica* was found to be the most sensitive to the action of the oxidizing agent. It is not entirely clear why BChl800 oxidation does not occur in this case (Figure 2 and Figure 3). In nonsulfur bacteria, both types of BChl are oxidized, but in smaller quantities. The most stable complex of all the studied bacteria was the LH2 complex from *Rba*. *sphaeroides*, a culture that has been used in classical works [1,26], and in *Mch.* *purpuratum*. These complexes contain spheroidene and okenone carotenoids, in contrast to other complexes, in which rhodopin and sometimes also anhydrorhodovibrin were the main carotenoids (Table S1). It is known that the oxidation of BChl in the pigment molecule results in the detachment of two protons at positions 7 and 8 in the second pyrrole ring and the formation of a double bond [23]. This is the only difference between AcChl and BChl molecules. The involvement of these protons in a hydrogen bond with amino acid residues of polypeptides can change their redox potential, and, as a consequence, hinder their oxidation (BChl800 in sulfur bacteria, both BChl in *Rba*. *sphaeroides* and *Mch.* *purpuratum*). It is clear that, for bacteria that have switched from autotrophic nutrition (sulfur bacteria are anaerobes or strict anaerobes) to heterotrophic nutrition and adapted to growth under conditions of different oxygen concentrations in the medium (nonsulfur bacteria), minimizing the possible oxidation of BChl is an important task. It is significant that, of all the bacteria studied, the behavior of the LH2 complex from *Rba*. *sphaeroides* is most consistent with the hypothesis that BChl is protected by carotenoids from oxidation by singlet oxygen, but it is not known whether this is the result of carotenoid activity or the formation of additional hydrogen bonds in the second pyrrole ring of BChl as another structural reason. This actually does not occur sometimes, and BChl in the LH2 complexes of certain bacterial species is easily oxidized in significant quantities. Despite the fact that a protective function of carotenoids is generally accepted, there are no quantitative estimates for its assessment. In practice, the main concept is based on the knowledge that in vitro (in a model system) carotenoids quench singlet oxygen with an efficiency of 90–100% [31,32]. Based on the results obtained in this work, we can fairly clearly speculate that BChl in the LH2 complexes containing 100% of the carotenoids is oxidized by singlet oxygen, and carotenoids in vivo (in LH2 complexes) are not able to effectively quench singlet oxygen, in contrast to the model systems.

**Figure 5:**
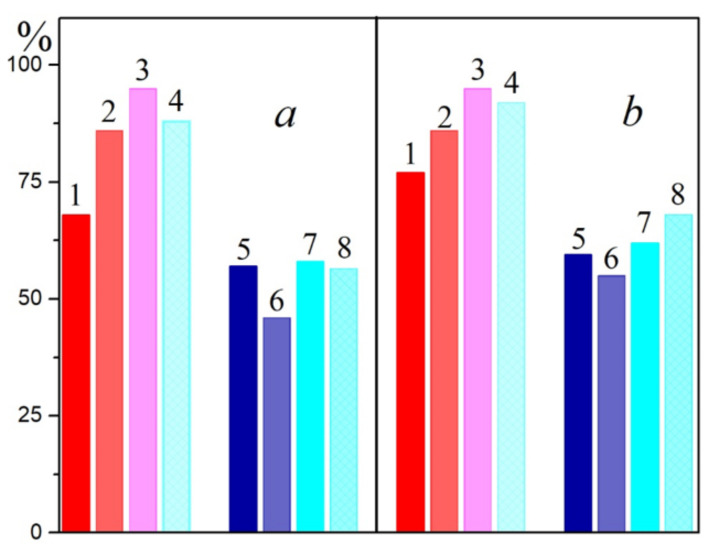
Diminution (%) of the long-wavelength Q_y_ band of BChl (BChl850/830) in the LH2 complex (**a**) and in the LH3 complex (**b**) from *T*. *sibirica* having different carotenoid contents (1—control; 2—80%; 3–40%; 4–10%) under illumination for 30 min in the presence of 50 μM RB (1, 2, 3, and 4) or blue light (5, 6, 7, and 8).

It is surprising that, with a decrease in the amount of carotenoids in the complexes, the efficiency of singlet oxygen during BChl oxidation not only does not increase, but, on the contrary, decreases (Figure 2a, Figure 3 and Figure 5). In the case of the LH2 complexes from *T*. *sibirica*, the most resistant were the samples that contained about 50% of the carotenoids (4–3 pigment molecules per complex). Such results are difficult to explain in light of modern concepts of the structure of such complexes. It can only be assumed that the removal of the carotenoid from their structure causes some changes in the latter, which complicates the access of singlet oxygen to BChl. These data coincide with the results of our work on carotenoidless samples of membranes from three types of bacteria [23,30]. In turn, they contradict the hypothesis of a protective role of carotenoids. It should be emphasized that studies on the direct identification of this function of carotenoids, as well as their interaction with singlet oxygen, on control and carotenoidless samples, have not been performed previously. Even in a pioneering work [1] devoted to the cultivation of the carotenoidless mutant *Rba*. *sphaeroides* in light under anaerobic conditions with subsequent transfer to aerobic conditions, only a decrease in the amount of BChl in the presence of oxygen was noted, and this effect was absent in control cells. According to the authors of that work, these results could be explained by the protective function of carotenoids. This hypothesis was immediately accepted by the scientific community and is now widely cited in articles and reviews [3,5,31,32]. However, for reasons unknown to us, the results of works [26,33], which were carried out in the same laboratory somewhat later than that work [1], using the same methods, mutants, and equipment, were not taken into account. The author of those works showed that: 1—the aeration of the mutant culture in the dark for 5 min with an air/CO_2_ mixture (95/5%) caused a delay in the destruction of the cells (in fact, a decrease in the amount of BChl) by 45 min; 2—the process of destruction of the mutant cells (in fact, a decrease in the amount of BChl) depended on temperature, and, when it was decreased from 30 to 20 °C, it slowed by ~25%, and at 1–6 °C, it stopped. The processes of the absorption of light quanta, the transition of pigment molecules to an excited state, the transfer of energy to oxygen, and its interaction with BChl do not have a lag phase and do not depend on the temperature in this range. The conclusions of the cited articles [26,33] correlate with the results of our work. We previously confirmed that BChl oxidation in the membranes of *Rba*. *sphaeroides* under the influence of light occurs only after the destruction of the LH2 complex and the appearance of monomeric BChl, which can generate singlet oxygen itself in the light [30]. Thus, our results directly show that carotenoids are not required to protect BChl from the action of singlet oxygen and that, with a decrease in the amount of these pigments in the complex, the resistance of BChl to the action of an oxidant increases. These results are in good agreement with the results of our earlier work in which we tried to assess the stability of control and carotenoidless complexes (from carotenoidless mutants or DPA-treated cells) of purple bacteria under red light illumination (λ > 700 nm, intensity 2000 W/m^2^). We found that the bleaching of the BChl850 band in LH2 complexes with or without carotenoids does not exceed 1–8% [34]. In that work, we first suggested that carotenoids are not required to protect BChl from photooxidation.

### 2.3. Generation of Singlet Oxygen by Carotenoids under Light in LH2 Complexes of Sulfur Bacteria

**Figure 6:**
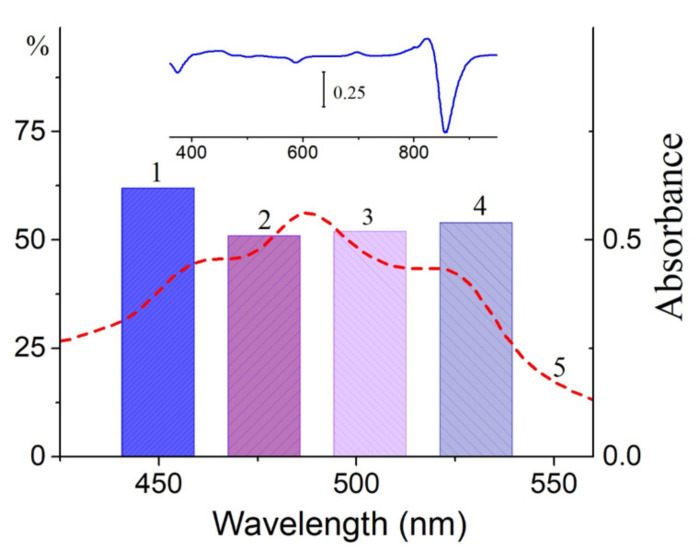
Diminution (%) of the long-wavelength Q_y_ band of BChl (BChl850) in the control LH2 complex from *Alc*. *vinosum* under illumination using bandpass filters: 1—440–465; 2—465–490; 3—490–515; 4—515–540 nm. For comparison, the absorption spectrum of the LH2 complex of *Alc*. *vinosum* in the carotenoid region (5) is shown. Inset: differential absorption spectrum of the LH2 complex from *Alc*. *vinosum*: “illumination for 30 min with filter 515–540 nm minus 0 min.”.

In this section, we would like to draw attention to the paradoxical, in our opinion, results obtained upon the illumination of LH2 complexes from sulfur bacteria with blue light absorbed by carotenoids. In the case of control complexes of LH2 with carotenoids from *Alc*. *vinosum*, this light caused the more efficient oxidation of BChl850 than RB did (Figure 1b). When most of the carotenoids (97–99%) from the LH2 complex were isolated from the DPA-grown cell, the two processes are almost the same (Figure 1b, curves 2 and 4). In LH2 and LH3 complexes from *T*. *sibirica*, blue light also oxidized BChl850/830 more efficiently than RB (Figure 2a,b). In fact, the changes recorded under the action of light and RB were identical (Figure 2c,d). A decrease in the amount of carotenoids in these complexes also increased the stability of the complexes in blue light (Figure 2). To be convinced of the participation of carotenoids in the BChl850 oxidation process, we recorded a simplified spectrum of the action of this process in the carotenoid region using bandpass filters (Figure 6), and the BChl850 photooxidation amplitude for all filters was quite close. Changes in the LH2 complex from *Alc*. *vinosum* are shown in Figure 6 (inset). Obviously, this process involves all the carotenoids present in the complex (rhodopin, didehydrorhodopin, etc.; Table S1).

It should be noted that the same decrease in the absorption of the BChl850 band can be caused by the illumination of LH2 complexes with blue light, and these changes are very similar to the action of singlet oxygen (Figure 1a, Figure 2c,d and Figure 6 inset). The effect of light in the carotenoid region for BChl850 oxidation in *Allochromatium (Alc.) vinosum* (formerly named *Alc*. *minutissimum*) was noted a long time ago in the study of the stability of BChl in membranes and complexes under illumination with light of different spectral regions (white, red, and blue) [35]. It was shown that the active component in the BChl photobleaching process was blue light absorbed by carotenoids. These results were initially perceived as an unusual effect with no explanation. Later, work in this direction was continued, and this made it possible to identify other bacteria (*Ectothiorhodospira* (*Ect*.) *haloalkaliphila, T*. *sibirica*, and *Thermochromatium tepidum*) in which the same oxidation of BChl850/830 was observed [36,37] (Bolshakov et al., in preparation). For example, this effect in the samples of *Ect*. *haloalkaliphila* was the same as that of *Alc*. *vinosum* [37,38]. Illumination by laser light with a wavelength of 532 nm (the region where BChl does not absorb) of LH2 complexes from *Ect*. *haloalkaliphila* also caused BChl850 oxidation [38].

Thus, we used two systems (Schemes (4) and (5)):

(BChl + Car) + (RB + hν) + O_2_ → ^1^O_2_* → AcChl (4)

BChl + (Car + hν) + O2 → ^1^O_2_* (?) → AcChl (5)

The oxidation of BChl850/830 in LH2 complexes of some sulfur bacteria under the action of singlet oxygen or blue light is probably the same process (Equations (4) and (5)). Based on these data, a reasonable assumption can be made that, under the action of blue light absorbed by carotenoids, singlet oxygen is generated in sulfur bacteria in LH2 complexes, and it oxidizes BChl850/830 to AcChl (Figure 2c,d and Figure 4). Our simplified experiment with bandpass filters shows that all carotenoids in the LH2 complex of Alc. vinosum can apparently participate in the oxidation of BChl850 as they form singlet oxygen under blue light illumination (Figure 5). The efficiency of carotenoids in our experiments was comparable to RB at a concentration of 100 μM.

One of the arguments for singlet oxygen participation in this process is its deceleration or interruption by the singlet oxygen quenchers. Earlier, we showed that well-known singlet oxygen quenchers (Trolox, sodium ascorbate, histidine, imidazole) easily interrupted the oxidation of BChl850 in the presence of BR [39]. Sodium azide was not active in these experiments. Similar negative results with sodium azide were also obtained in the system “reaction centers photosystem 2/BR” [40]. Additional experiments on the effect of singlet oxygen quenchers on the BChl850 photooxidation process in LH2 complexes under blue light were carried out, and it was shown that Trolox and sodium ascorbate effectively interrupt this process, in contrast to histidine (Figure S5). We assume that the absence of the histidine effect is due to the fact that it is not a penetrating agent, but the processes of singlet oxygen formation and BChl oxidation occur inside the LH2 complex where carotenoids and BChl are located.

**Figure 7:**
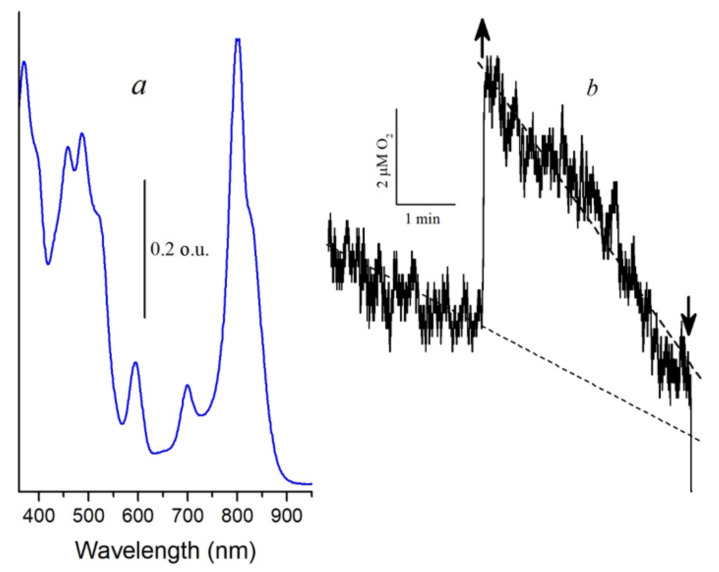
(**a**), Absorption spectra of LH2 complexes from *Alc*. *vinosum* after illumination for 60 min under blue light; (**b**), Histidine (20 mM) catalysed light-dependent oxygen uptake by isolated LH2 complexes from *Alc vinosum* with the preliminary bleached B850 band (**a**). Arrow up and down—light on and off, respectively.

Another argument for proving the participation of singlet oxygen in the oxidation of BChl850 is the measurement of the oxygen concentration in the cell using the Clark electrode. This technique is widely used in biological systems to determine the generation of singlet oxygen [40,41]. In the control complexes, the formation of singlet oxygen using the Clark electrode was not detected, which is associated with its consumption for BChl850 oxidation (results not shown). After preliminary photooxidation of BChl850 under illumination for 60 min with blue light (Figure 7a), we managed to exclude this process and register in these complexes a small signal of oxygen absorption in the presence of histidine by the Clark electrode, which indicates the generation of singlet oxygen (Figure 7b). These results correlate well with the data we obtained using the probe Singlet Oxygen Sensor Green. It is transformed into a fluorescent endoperoxide form when singlet oxygen is bound. The formation of singlet oxygen in the membranes of bacteria (*Alc. vinosum*, mutant *Rb. sphaeroides* G1C, *Rba. blasticus* K-1, and *Rps. faecalis*) was noted under white light illumination by this method. The red light absorbed by BChl was not active in this process. Therefore it was suggested that the carotenoids of these bacteria are involved in the generation of singlet oxygen [42].

It is not entirely clear why BChl does not participate in the generation of singlet oxygen, since the pathway: carotenoid singlet → BChl singlet → BChl triplet → singlet oxygen—seems to be the most probable according to modern concepts of the excitation energy transfer in photosynthetic bacteria. However it should be noted that in our experiments, we have never recorded the participation of BChl in the generation of singlet oxygen (BChl850 photooxidation). Note that we have recently begun work on measuring the action spectrum of the BChl850 photooxidation process using a laser with a variable wavelength (440–870 nm) and preliminary results show the activity of carotenoids in this process and the absence of BChl participation in it (Proskuryakov et al., unpublished).

A reasonable question arises: why the ability of carotenoids to generate singlet oxygen has not yet been established. Perhaps this is due to a coincidence of circumstances: first, in model systems (solvents), carotenoids perfectly quench singlet oxygen, and no one saw the need to look for a completely opposite function of these pigments in vivo (according to our preliminary data, carotenoids generate singlet oxygen in model water systems with low quantum yield; Bolshakov et al., preparing for publication); second, BChl efficiently generates singlet oxygen in vitro. Therefore, it was suggested that this property can be partially preserved in vivo. However, we could not find results on measuring the generation of singlet oxygen by carotenoid-free mutants, in which this process, according to the modern concept, should proceed with high efficiency. Third, the main bacteria studied in most laboratories are nonsulfur bacteria. The effect of BChl850 oxidation by singlet oxygen or blue light is very weak or absent in these bacteria.

Nevertheless there has been some recent progress in this direction. It was established that the direct generation of singlet oxygen by phytofluene (the first carotenoid in the biosynthetic chain) in a model system occurs with a quantum yield close to 1 [43]. Recently, it was suggested that ζ-carotene (the second carotenoid in the biosynthesis chain) has a higher level of the triplet state and therefore, can interact with oxygen to form singlet oxygen [25]. These works only consider the triplet–triplet interaction between carotenoid and oxygen. Note that we have obtained new data that neurosporene is capable of generating singlet oxygen with a low quantum yield and is very active in the photooxidation of BChl850 (Moskalenko, Krasnovsky, et al., preparing for publication). Spheroidene, a carotenoid from nonsulfur bacteria, is also active in the process of BChl850 photooxidation (Moskalenko et al., preparing for publication).

Note one more unique feature of the light-harvesting complexes LH2/LH3 from some sulfur bacteria—they are natural sensors that can determine the presence of singlet oxygen in an aqueous medium by decreasing the absorption band of BChl850/830 and the appearance of the band of an oxidized product, AcChl. For example, our preliminary data showed the BChl850 band bleaching (generation of singlet oxygen) in the system “reaction center of photosystem 2/light-harvesting complex LH2” under illumination at 670 nm (red band of reaction center of photosystem 2) (Moskalenko et al., in progress). We hope that our results will stimulate research towards the mechanism of singlet oxygen release by carotenoids using precise physical methods.

Based on all these data, a reasonable assumption can be made that, under the action of blue light absorbed by carotenoids, singlet oxygen is generated in LH2 complexes of sulfur bacteria and oxidizes BChl850/830 to AcChl. Perhaps this is true for carotenoids with 9–11 double bonds.

## 3. Experimental Section

**Figure 8:**
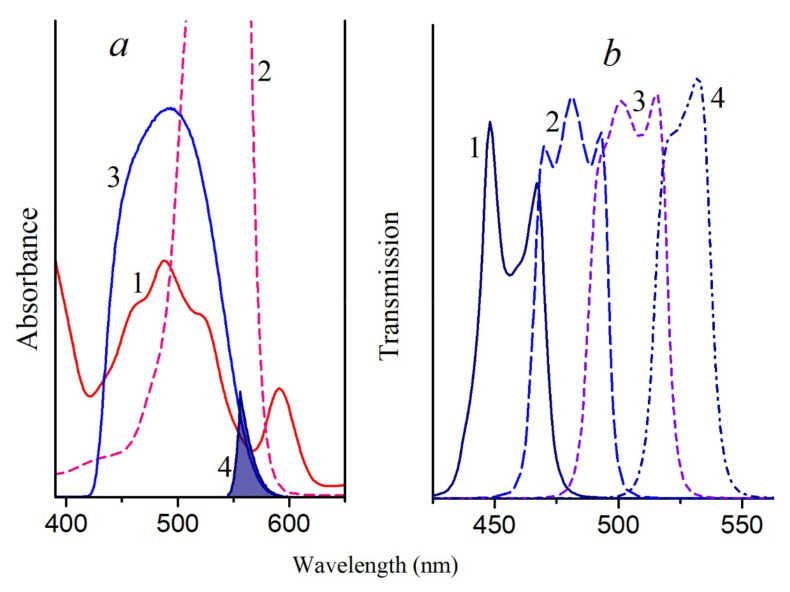
(**a**)—Absorption spectra of *Alc. vinosum* membranes (1) and RB (2), as well as the transmission spectra of the combination of light filters SZS-22 + ZhS-12 (3) (blue light) and SZS-23 + OS-13 (4); (**b**)—transmission spectra of bandpass light filters: 1—440–465; 2—465–490; 3—490–515; 4—515–540 nm.

LH2 complexes isolated from the membranes of photosynthetic bacteria: *Allochromatium (Alc.) vinosum* (formerly named *Alc*. *minutissimum*) was obtained from the collection of the Department of Microbiology, Moscow State University); *T*. *sibirica*, *Rba*. *sphaeroides*, *Rbl*. *acidophilus*, *Rps*. *rubrum*, *Mch. purpuratum*, and *Rps*. *palustris* were also studied. All these bacteria contain LH2 complexes (B800–850) (with the exception of *T*. *sibirica* cells, in which an additional LH3 complex (B800–830) is present), and *Rps*. *rubrum* contains only the LH1 complex. The methods for isolating the complexes have been described in detail earlier [19,20,21], and for their isolation, we used the detergent dodecylmaltoside and Tris–HCl buffer at pH 7.5. The isolation of complexes with a reduced content of carotenoids was performed from cells grown in the presence of diphenylamine as described in the literature [19,20,21,22]. If the sample contained only traces of carotenoids, then it was designated as a DPA sample; otherwise, the amount of carotenoids in the sample is indicated as a percentage. Rose Bengal (RB) was used as a source of singlet oxygen; it masks the absorption region of carotenoids (440–580 nm) but does not overlap the absorption in the region above 630 nm, which makes it possible to register the appearance of the BChl oxidation product (~700 nm) spectrally (Figure 8a). The concentration of RB used was 50 μM; this concentration was selected so that the RB effect was comparable to the effect of the illumination of samples from sulfur bacteria with blue light. Two standard light filters (SZS-23 + OS-13) were combined for illuminating the samples containing RB. This filter combination cuts a narrow region of ~550–580 nm where BChl and carotenoids have low absorption (Figure 8a). Under these conditions, the rate of singlet oxygen generation by RB was about 110 μM per minute. Control samples (without RB) were illuminated under the same conditions. These results were taken into account in the formation of the BChl850 oxidation curves. All illumination experiments were carried out at 24 °C in a thermostatic cell. Complexes were irradiated in the entire area of carotenoids with blue light (filters SZS-22 + ZhS-12,) and in narrower ranges using bandpass filters (440–465, 465–490, 490–515, and 515–540 nm; Figure 8b) produced by OOO “Photooptic”, Obninsk, Russia. The measurement error in all experiments did not exceed 5–10%.

Light-induced oxygen uptake in LH2 complexes from *Alc. vinosum* was measured in a temperature-controlled chamber using a Clark-type electrode as described in [42]. The samples were illuminated with blue light (filters SZS-22/ZhS-12, 30 W per m^2^) using a halogen lamp (500 W, 220 V) and a fiber optic light guide with a diameter of 1 cm.

HPLC analysis of pigment extracts from control and irradiated samples in the presence of RB was performed as described in the literature [22]. Carotenoids, BChl, and its oxidation products were identified by their retention times on the column and by the absorption spectrum using the LCsolution software (Shimadzu). Absorption spectra were recorded on a Cary 50 spectrophotometer (Agilent Technology, Santa Clara, CA, USA).

## 4. Conclusions

Summarizing the results of this work, we can draw the following conclusions: 1—carotenoids do not protect BChl from destructive interactions with singlet oxygen in LH2 complexes from purple photosynthetic bacteria; 2—Carotenoids, upon excitation with blue light, are capable of generating singlet oxygen, which oxidizes BChl850 to AcChl in LH2 complexes from some sulfur bacteria. The mechanism for this generation has not yet been established.

## Supplementary Materials

The following are available online, Figure S1: Absorption spectra of control (1) and DPA (2) LH2 complexes of Alc. vinosum. Figure S2: Absorption spectra of LH2 complex (a) and LH3 complex (b) from T. sibirica with different carotenoid content (1—control; 2—80%; 3—40%; 4—10%), Figure S3: HPLC of pigments of the LH2 complex (a) and the LH3 complex (b) of T. sibirica after illumination for 30 min in the presence of 50 μM RB. Peak identification: 1–5—BChl and its derivatives; 6—3,4-didehydrorhodopin + AcChl; 7—rhodopin; 8—spirilloxanthin; 9, 10—AcChl derivatives; 11—anhydrorhodovibrin. The circles mark the absorption bands Qy of AcChl. Figure S4: Absorption spectra of carotenoids and AcChl from HPLC of LH3 complex (b) from T. sibirica: 6—3,4-didehydrorhodopin + AcChl; 7—rhodopin; 8—spirilloxanthin; 9, 10—AcChl derivatives; 11—anhydrorhodovibrin. Table S1: The main carotenoids in LH2/LH3 complexes, Figure S5: Absorption spectra of control LH2 complexes of Alc. vinosum before (1) and after illumination blue light for 30 min in the presence of: 1 mM sodium ascorbate (2), 100 μM Trolox (3),10 mM histidine (5) and without additives (4).

## Sample Availability

Some samples of the LH2 complexes are available from the authors.
