# Supplementary material for: Carotenoids Do Not Protect Bacteriochlorophylls in Isolated Light-Harvesting LH2 Complexes of Photosynthetic Bacteria from Destructive Interactions with Singlet Oxygen
Source: Molecules. 2021 Aug 24;26(17):5120. doi: 10.3390/molecules26175120 (PMC8434301; doi:10.3390/molecules26175120)
Supplement: Supplementary file 1 [file molecules-26-05120-s001.zip › molecules-1272376-supplementary.pdf]

Carotenoids Do Not Protect Bacteriochlorophylls in Isolated Light-Harvesting LH2 Complexes of Photosynthetic Bacteria from Destructive Interactions with Singlet Oxygen

Makhneva Z.K., Bolshakov M.A., Moskalenko A.A.*
Institute of Basic Biological Problems RAS, Pushchino, 142290, Russia

*correspondence: andrey-moskalenko@rambler.ru

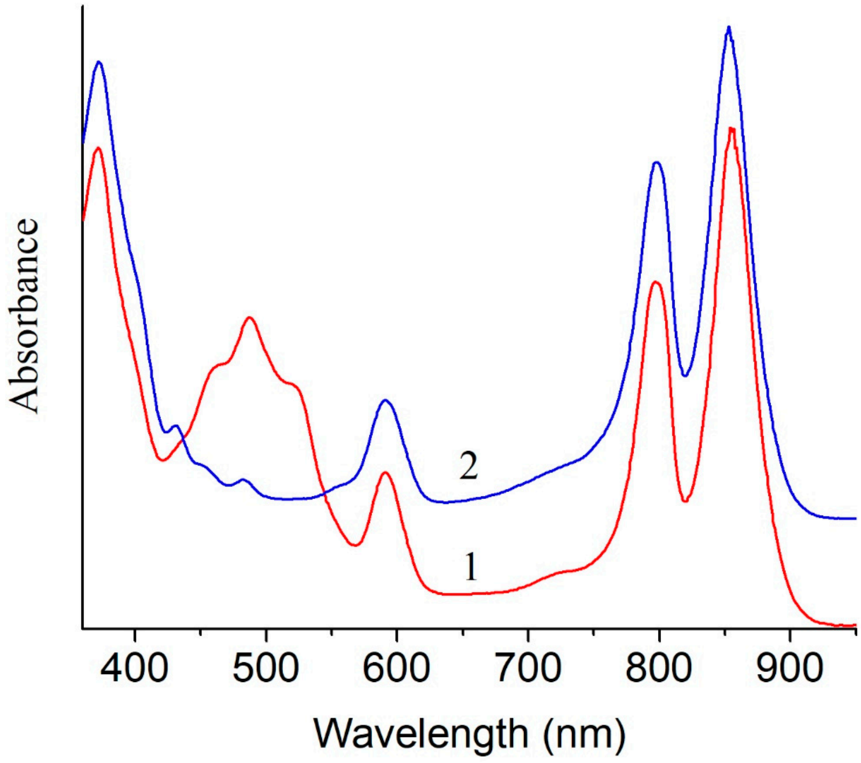

Figure S1 Absorption spectra of control (1) and DPA (2) LH2 complexes of *Alc. vinosum*.

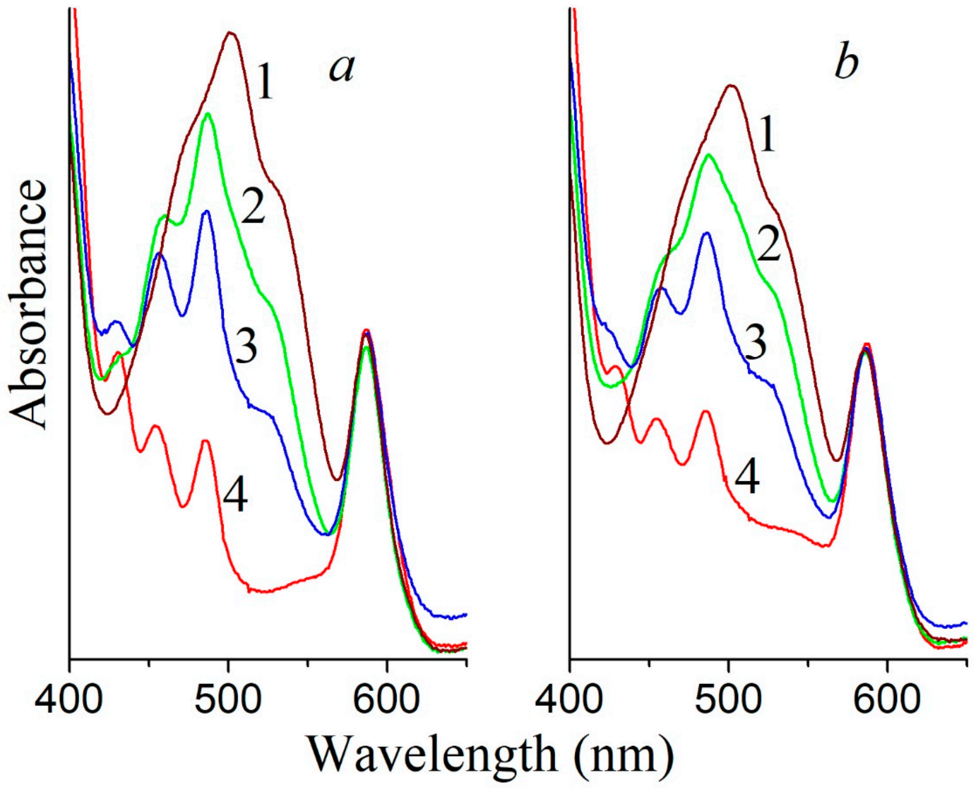

Figure S2. Absorption spectra of LH2 complex (a) and LH3 complex (b) from *T. sibirica* with different carotenoid content (1 – control; 2 – 80%; 3 – 40%; 4 – 10%).

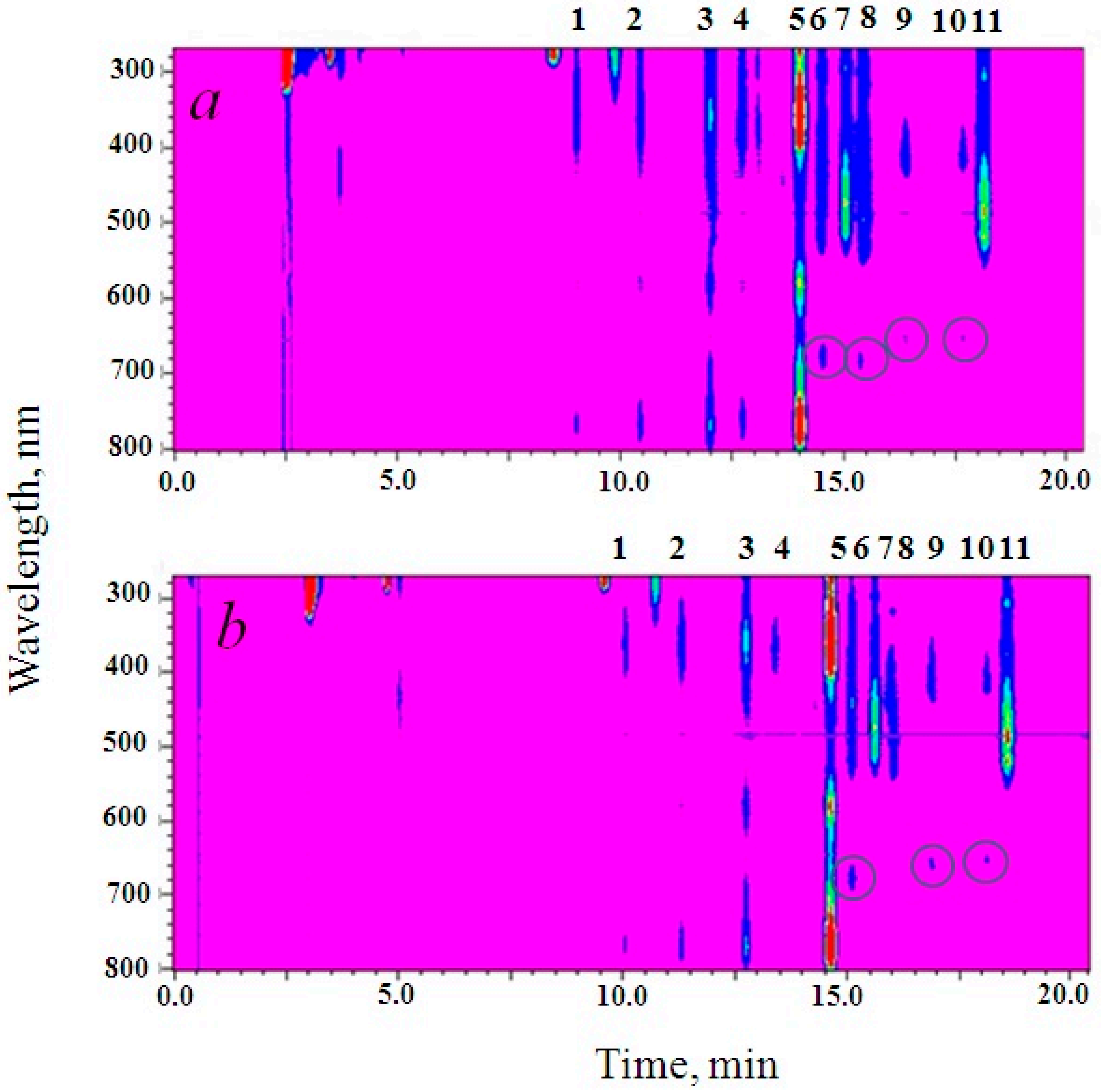

Figure S3. HPLC of pigments of the LH2 complex (a) and the LH3 complex (b) of *T. sibirica* after illumination for 30 min in the presence of 50 μM RB. Peak identification: 1-5 – BChl and its derivatives; 6 – 3,4-didehydrorhodopin + AcChl; 7 – rhodopin; 8 – spirilloxanthin; 9, 10 – AcChl derivatives; 11 – anhydrorhodovibrin. The circles mark the absorption bands Q_y of AcChl.

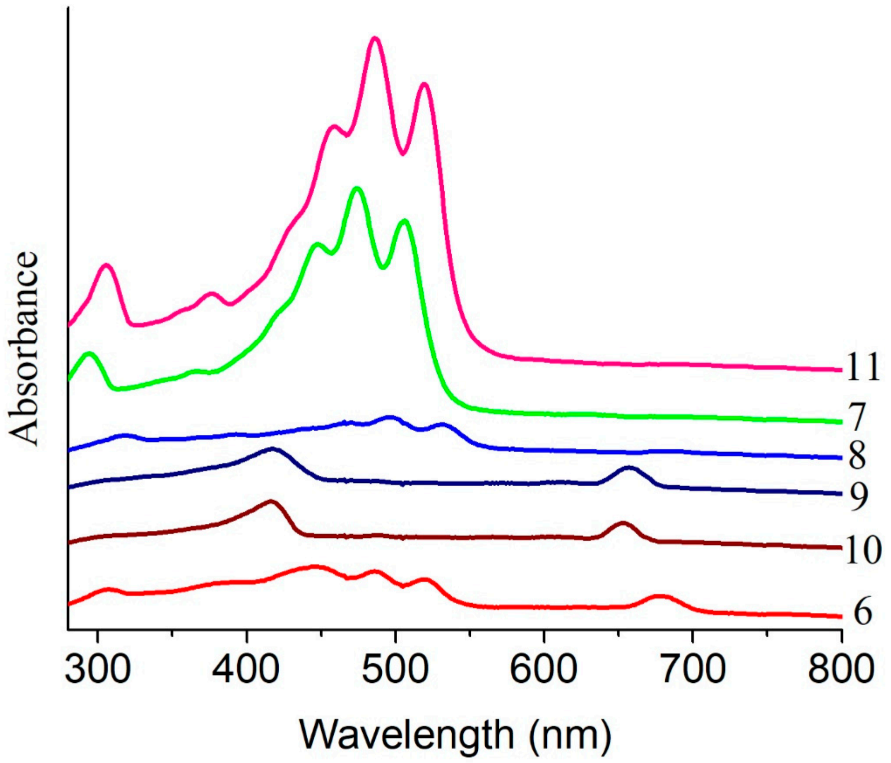

Figure S4. Absorption spectra of carotenoids and AcChl from HPLC of LH3 complex (b) from *T. sibirica*: 6 – 3,4-didehydrorhodopin + AcChl; 7 – rhodopin; 8 – spirilloxanthin; 9, 10 – AcChl derivatives; 11 – anhydrorhodovibrin.

Table S1. The main carotenoids in LH2/LH3 complexes [a].

carotenoid	*Alc. vinosum* LH2		*T. sibirica* LH2/LH3								*Rps. palustris* LH2	*Rba. sphaeroides* LH2	*Rbl. acidophilus* LH2	*Mch. Purpuratum* LH2
	100% CAR	DPA <5% CAR [b]	100% CAR		80% CAR		40% CAR		10% CAR[b]		100% CAR	100% CAR	100% CAR	100% CAR
			LH2	LH3	LH2	LH3	LH2	LH3	LH2	LH3				
ζ-Carotene and its derivatives		**3,5**							**4**	**3**				
Neurosporene and its derivatives		**1**			**9,5**	**14**	**29**	**22**	**5**	**3,5**				
Lycopene							**14**	**13**			**21**		**11**	
Rhodopin	**73**		**31**	**31**	**43**	**31,5**		**6,59**			**52**		**82,5**	
3,4-Didehydrorhodopin	**19**										**23**			
Anhydrorhodovibrin			**58,5**	**53,5**	**21**	**22**								
Spirilloxanthin				**10**										
spheroidene												**96,5**		
Okenone														**95**

[a] Main carotenoids (CAR) with concentrations exceeding 6%. [b] For samples with a low carotenoid content, the main pigments are given taking into account free carotenoid pockets [22].

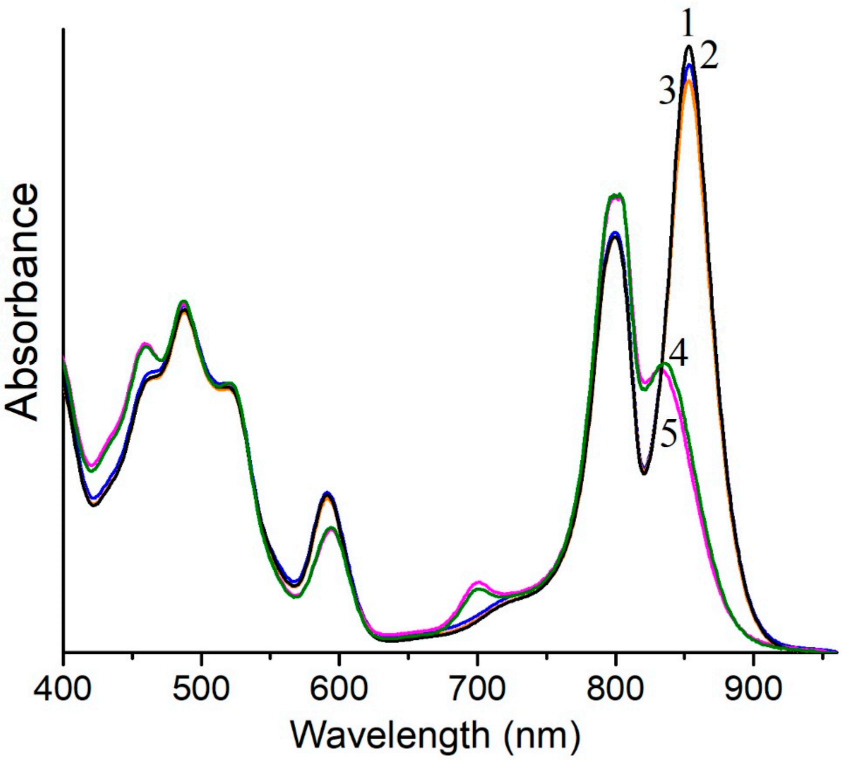

Figure S5. Absorption spectra of control LH2 complexes of *Alc*. *vinosum* before (1) and after illumination blue light for 30 min in the presence of: 1 mM sodium ascorbate (2), 100 µM Trolox (3),10 mM histidine (5) and without additives (4).
